# Echoes from the past: Regional variations in recovery within a harbour seal population

**DOI:** 10.1371/journal.pone.0189674

**Published:** 2018-01-03

**Authors:** Sophie M. J. M. Brasseur, Peter J. H. Reijnders, Jenny Cremer, Erik Meesters, Roger Kirkwood, Lasse Fast Jensen, Armin Jeβ, Anders Galatius, Jonas Teilmann, Geert Aarts

**Affiliations:** 1 Wageningen Marine Research, Wageningen University & Research, Den Helder, the Netherlands; 2 Aquatic Ecology and Water Quality Management, Wageningen University, Wageningen, the Netherlands; 3 Fisheries and Maritime Museum, Esbjerg, Denmark; 4 Landesbetrieb für Küstenschutz, Nationalpark und Meeresschutz Schleswig-Holstein Nationalparkverwaltung, Tönning, Schleswig-Holstein, Germany; 5 Department of Bioscience, Aarhus University, Roskilde, Denmark; Universidade de Aveiro, PORTUGAL

## Abstract

Terrestrial and marine wildlife populations have been severely reduced by hunting, fishing and habitat destruction, especially in the last centuries. Although management regulations have led to the recovery of some populations, the underlying processes are not always well understood. This study uses a 40-year time series of counts of harbour seals (*Phoca vitulina*) in the Wadden Sea to study these processes, and demonstrates the influence of historical regional differences in management regimes on the recovery of this population. While the Wadden Sea is considered one ecologically coupled zone, with a distinct harbour seal population, the area is divided into four geo-political regions i.e. the Netherlands, Lower Saxony including Hamburg, Schleswig-Holstein and Denmark. Gradually, seal hunting was banned between 1962 and 1977 in the different regions. Counts of moulting harbour seals and pup counts, obtained during aerial surveys between 1974 and 2014, show a population growth from approximately 4500 to 39,000 individuals. Population growth models were developed to assess if population growth differed between regions, taking into account two Phocine Distemper Virus (PDV) epizootics, in 1988 and 2002 which seriously affected the population. After a slow start prior to the first epizootic, the overall population grew exponentially at rates close to assumed maximum rates of increase in a harbour seal population. Recently, growth slowed down, potentially indicative of approaching carrying capacity. Regional differences in growth rates were demonstrated, with the highest recovery in Netherlands after the first PDV epizootic (i.e. 17.9%), suggesting that growth was fuelled by migration from the other regions, where growth remained at or below the intrinsic growth rate (13%). The seals’ distribution changed, and although the proportion of seals counted in the German regions declined, they remained by far the most important pupping region, with approximately 70% of all pups being born there. It is hypothesised that differences in hunting regime, preceding the protection in the 1960’s and 1970’s, created unbalance in the distribution of breeding females throughout the Wadden Sea, which prevailed for decades. Breeding site fidelity promoted the growth in pup numbers at less affected breeding sites, while recolonisation of new breeding areas would be suppressed by the philopatry displayed by the animals born there. This study shows that for long-lived species, variable management regimes in this case hunting regulations, across a species’ range can drive population dynamics for several generations.

## Introduction

Throughout history, humans have impacted wildlife populations. Initially, main impacts resulted from hunting and fishing for food and resources. Later, culling was also carried out to protect livestock, crops, game, or fish stocks. As the human population grew, so did the intensity of hunting, habitat destruction, pollution and effects on global climate, leading to fundamental changes in animal populations [1–4]. The combined and often synergistic effects of these threats [5] render it complicated to identify the particular drivers for an observed change. This includes that the compromising physiological stress exerted by these threats could make the populations susceptible to e.g. emerging infectious diseases both in terrestrial and marine ecosystems [6–8]. It is therefore not always clear why efforts to protect species and biodiversity [9–13], succeed or fail [14, 15].

Hunting, both for subsistence and commerce or as a result of local bounties, was the main threat to seal populations until the second half of the 20^th^ century, resulting in a gradual ban throughout most of Europe [16–24]. For harbour seals, pollution and disturbance as a result of industrialisation and urbanisation, as well as virus epizootic events, further affected population development [3, 25–28]. Recently, British harbour seal populations have suffered new decreases for which the causes are uncertain [29–31], while in Southern Scandinavia and the Wadden Sea, harbour seal populations have shown recovery [32, 33].

**Fig 1:**
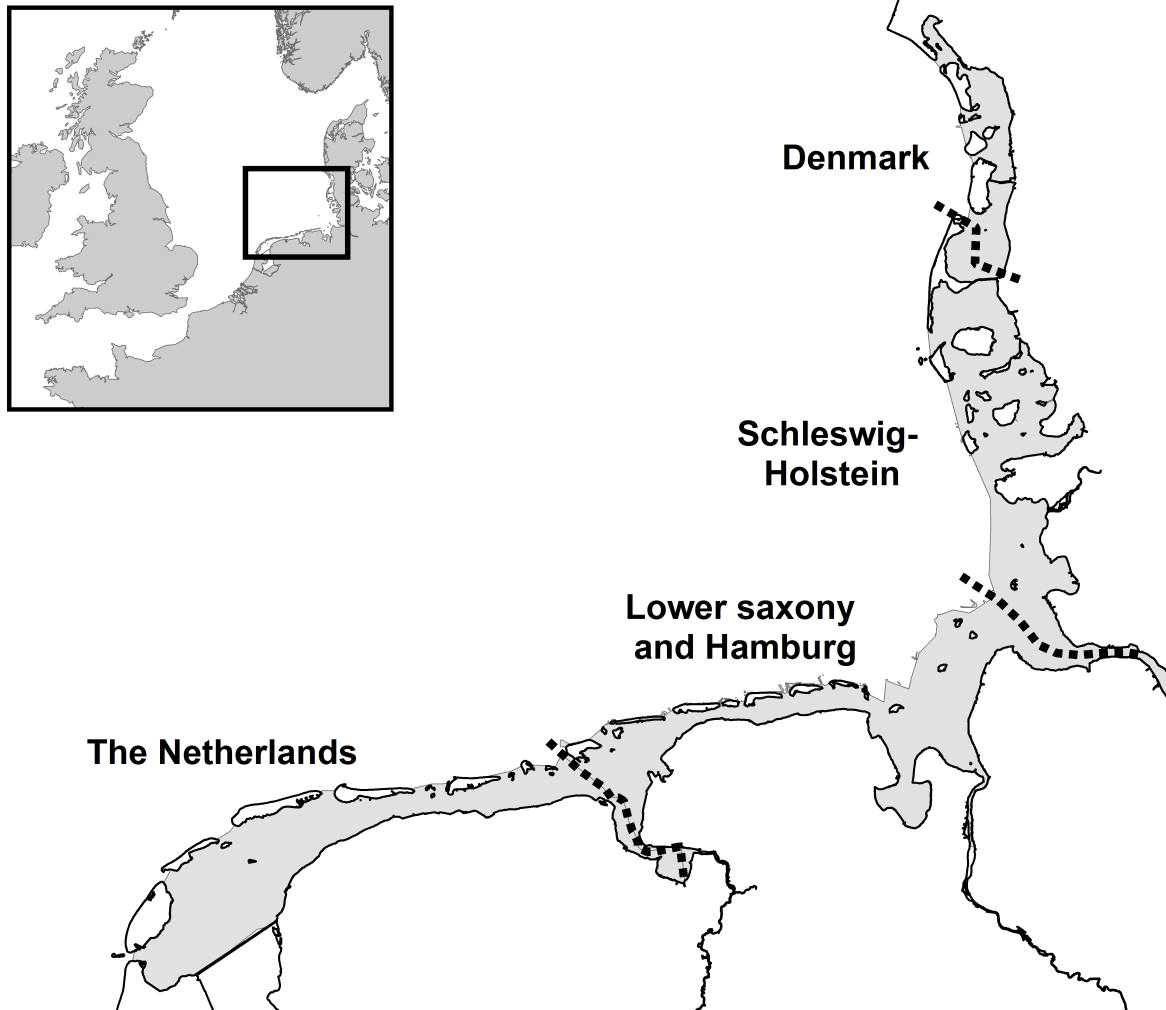
Map of the international Wadden Sea (grey area) dotted black lines indicate the borders between regions. Inlay: the North Sea situating the study area.

Harbour seals in the international Wadden Sea, between Den Helder in the Netherlands and Skallingen north of Esbjerg in Denmark, are considered a distinct population based on their genetic difference from seals in neighbouring regions in the North Sea area [34, 35]. There are four management regions for the Wadden Sea: the Netherlands (NL), Lower Saxony and Hamburg (Germany; LS), Schleswig-Holstein (Germany; SH), and Denmark (DK) (Fig 1). Despite challenges caused by virus epizootics and growing anthropogenic use of their habitat in the past 50 years, the Wadden Sea harbour seal population has shown exceptional recovery after being severely depleted by hunting. The close cooperation between these regions to monitor the development of this population since 1974 provides a unique dataset to study the population as a whole, but also to study regional differences in the population development and the factors controlling them.

Around 1900, the harbour seal population size in the Wadden Sea might have been at least 40,000 animals [23], despite enduring centuries of hunting [22, 23, 36]. Hunting pressure increased in the early 20^th^ century due to the more intensive use of fire arms, and seal numbers dropped dramatically to approximately 8000 harbour seals in 1960 [23]. As a response to the low numbers, seal hunting was gradually prohibited: first in the Dutch Wadden Sea in 1962, followed by Lower Saxony in 1971, Schleswig-Holstein in 1973, and finally the Danish Wadden Sea in 1976 [11, 37]. Despite the ban, numbers continued to drop and by 1974, counts in the international Wadden Sea were down to less than 4000 animals [3]. Up to the 1980’s, recovery was hindered by the low reproduction especially in the Netherlands, as a result of pollution by polychlorinated biphenyls (PCBs) [28]. Still, a slow recovery could be observed throughout the Wadden Sea. Then in 1988, an outbreak of Phocine Distemper Virus (PDV) killed over 50% of the Wadden Sea population [38] and, as the population had recovered, a second outbreak of PDV struck in 2002, killing approximately the same proportion of the population [39, 40]. Even with these set-backs, the population continued to grow, and in 2015 the population size in the international Wadden Sea was estimated at 39,000 animals [41], approximately the same amount that were thought to be present in 1900 [23].

The very low numbers after the first PDV epizootic in 1988, gave rise to the protection of harbour seals in Europe under the Habitat and Bird Directive of the EU (appendix II), and since 1991 the Wadden Sea harbour seals have been protected by an agreement under the Convention on the Conservation of Migratory Species of Wild Animals [42, 43] concluded between the Wadden Sea countries (Denmark, Germany and the Netherlands). This agreement is enforced by means of a Trilateral Seal Management Plan. A basis for management is the close cooperation between the countries in the Trilateral Seal Expert Group (TSEG), which strives, for example, to maintain the annual synchronised monitoring of the whole population by aerial surveys used to fine-tune trilateral or local management decisions.

This study represents one of the few long-term (40-year) animal population studies where management differed regionally, providing insight in factors affecting population trends and pup production in the processes of recovery from severe overexploitation. Results potentially have implications for successful conservation of long-lived, broad-ranging, species and the ecosystems in which they live.

## Material and methods

### Data collection

Harbour seals in the Wadden Sea were counted by aerial survey techniques annually over a 40-year period (1974–2014; S1 and S2 Tables). Aerial surveys were carried out from fixed-wing aircraft, flying at elevations of 500–1000 ft. (150–300 m) and speeds of 160 to 220 km/h. Surveys were conducted within a 4-h window between 2 h before and 2 h after low tide, on days when low tides occurred between 12:00 and 16:00 local time [44]. Surveys were performed on days with no or little rainfall (<10 mm precipitation, measured between 08:00 UTC the preceding day and 08:00 UTC of the flight day), and winds generally were below 25 knots. Prior to the mid 1990’s, seals were counted directly by the observers during the flight in all regions, but from then onwards in Denmark, Schleswig-Holstein and the Netherlands seals were photographed using camera with slide film (until 2000) or digital camera (from 2000 onwards). The animals were counted by the regional monitoring groups, from the pictures. In Lower Saxony, observers continued to count directly during the flight. The objective was to survey each geopolitical region (Denmark, Schleswig-Holstein, Lower Saxony including Hamburg and the Netherlands: Fig 1) completely at least five times per year: at least three times during the pupping period (June/July) and at least twice during the moult period (August). The international teams aimed to survey on the same dates, but local circumstances sometimes led to changes or cancellation of flight dates. While data of the individual surveys were available for most years, only the maximum pup and maximum moult counts were available for Germany and Denmark in the first period (1974–1987). Permits to fly at the requested altitude above the Wadden Sea were issued separately in the four regions by the following authorities: Province of Friesland (for the Dutch Wadden Sea), Niedersächsisches Landesbehörde für Straßenbau und Verkehr (Lower Saxony), Landesbetrieb Straßenbau und Verkehr Schleswig-Holstein (Schleswig-Holstein) and Danish Transport, Construction and Housing Authority (Denmark)

During the pupping season, harbour seal pups can be discerned from older animals based on their coloration, size and often proximity to a larger seal (a mother). During the annual moult, shortly after the breeding period, however, pups cannot be discerned from yearlings and, hence, only total seal numbers were recorded. Because of the lack of dimorphism in the species, it was not possible to distinguish males from females during surveys. Grey seals recolonised the Wadden Sea in the late 1980’s [45–47] and were distinguished from harbour seals based on their habit to lie in clusters, generally larger size, shape (elongated head, often broader thorax), and colouration (e.g. larger spots), and depending on the season, their moult status, as the two species moult at different times of the year. Single young grey seals lying amongst large groups of harbour seals might not have been recognised, but it is unlikely that these individuals compromised the accuracy of estimates of number of either species.

### Data processing

Count data were used to obtain population growth rates for the four Wadden Sea regions and to estimate proportion of pups. All data, including flight conditions and additional notes, were combined into a database for further analysis. Records were allocated to a period, based on the occurrence of the two PDV epizootics: 1974–1987 (I); 1989–2001 (II); and 2003–2014 (III). Data collected in the years of the virus outbreaks (i.e. 1988 and 2002) were excluded from our analysis as the outbreaks occurred during the monitoring period and biased the counts. For Lower Saxony in 1996 and 2008, no counts were available so, instead, numbers were estimated based on the trend in the counts [48].

Assuming that in most years the peak in the number of pups was captured at least once during the three to five surveys, the response variable for the pups was defined as the annual maximum number of pups counted in each region. The numbers recorded during the peak in pup numbers represent approximately 70% of the total annual pup production [38, 49–51].

The moult counts (including animals of all age classes) are often used as an index of the total population size [52]. During the moult, numbers of animals hauled out on the sandbanks show a less clear peak than the pupping peak. This is because they represent the sum of different age classes that haul-out in different proportions in relation to timing of their moult [53]. For the German and Danish regions during period I (1974–1987), only maximum moult counts were available. Therefore maximum count during moult was used as response variable.

### Population growth rate models

Exponential and density-dependent growth models were fitted to both the pup and moult data. To estimate the exponential growth, generalized linear models (GLM) were fitted, assuming a negative binomial error distribution for the annual pup and moult estimates. The exponential growth model was defined as:

$$N_{t}=\exp\left(\beta_{0}+rt\right)$$ Eq 1

where N is either the estimated annual pup or moult count, t is the year (t_0_ = 1974), exp(β_0_) is the initial estimated count at t = 0, and r is the instantaneous rate of increase. The initial analysis was performed on the total Wadden Sea population (the sum of all regions). The simplest model included an intercept and year t as an explanatory variable, i.e. assuming a continuous exponential growth between 1974 and 2014. This model was subsequently expanded by allowing the height (defined by the GLM intercept β_0_) and growth rate (defined by the GLM slope parameter r) to vary between the periods (I, II, and III). Subsequently, new models were fitted to the regional count data, allowing the height and growth rate to also vary between the four regions (NL, LS, SH & DK) and periods, and with interactions between these. The density-dependent model was defined as:

$$N_{t}=\frac{\kappa }{1+\exp\left(a-rt\right)}$$ Eq 2

where K is the carrying capacity parameter, a is the height and r the growth rate. As for the exponential models, the density-dependent models were first fitted to the total Wadden Sea counts. The simplest model included single estimates for *K*, *a* and *r*. Next, similar to the exponential model fitting, the models were extended by allowing a and/or r to vary by period. Finally, density-dependent models were fitted to the survey data by region, and a separate K for each region was estimated. These density-dependent models were fitted using generalized non-linear models GNM (R-package “GNM” [54]). A GNM is similar to a generalized linear model, but it also allows for the estimation of parameters of non-linear models, like Eq 2. Fitting the model of Eq 2 using GNM produces maximum likelihood estimates for the parameters *K*, *a* and *r*. The response variable (i.e. counts) was assumed to follow a negative binomial distribution, hence allowing for over-dispersion.

The Akaike Information Criteria (AIC) [55] was used to select the best model. All analysis were carried out in the software R [56].

Finally, we estimated proportion of pups [57] for each region. We defined the proportion of pups as the maximum number of pups observed each year divided by the number of seals observed during the moult surveys [38].

## Results

### Population developments

**Fig 2:**
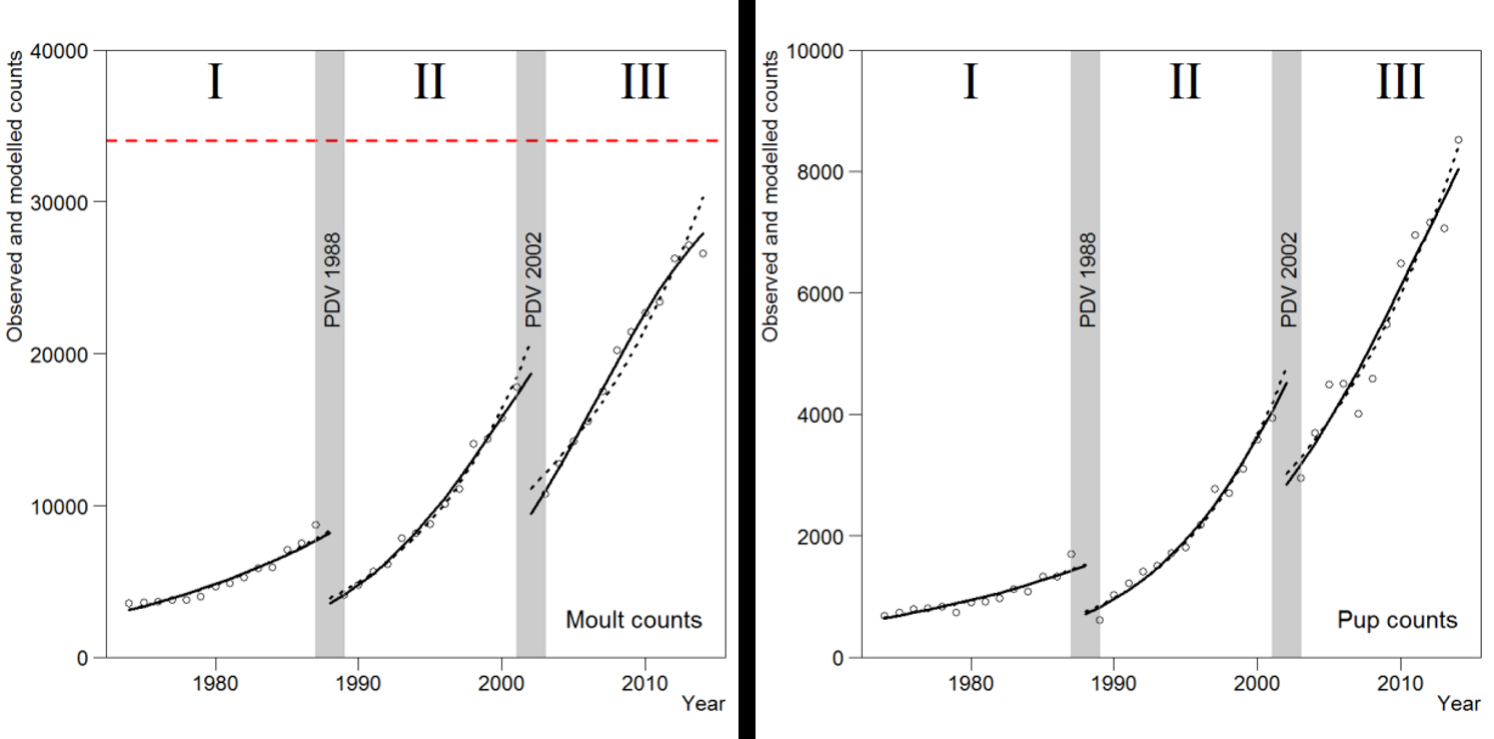
Observed (points) and modelled (lines) counts for the total Wadden Sea population during the years 1974-2014.Dashed lines represent the best fitting exponential models (i.e. interaction between year and period), and solid lines represent density-dependent models (also interaction between year and period). The horizontal dashed red line indicates the estimated carrying capacity for the moult counts.

Despite the occurrence of the two PDV-epizootics in 1988 and 2002, the number of seals during the moult counts for the whole Wadden Sea grew considerably during the study period (Fig 1 and Table 1). In the pre-epizootics period (period I), they increased from 3,571 in 1974 to 8,670 in 1987, equivalent to an annual rate of 7.2% (95% CI: 6.4%-8.1%; Table 2). The density-dependent model estimated that the number of moulting animals in 1988 declined from 8,200 to 3,600, a drop of 56%, while the exponential model estimated a drop of 54%. After this first PDV, the seals recovered during period II and counts reached pre-epizootic levels by 1995, and then grew to 16,738 animals in 2001. The annual rate of increase in period II was 12.7% (95% CI 11.7%-13.8%). Again in 2002 the PDV epizootic decimated the population and counts were down to 10,285 in 2003, equivalent to 50% and 47% for the density-dependent and exponential models, respectively. The population recovered and reached pre-epizootic levels by 2007, then grew to a count of 23,722 in 2014. The annual rate of increase of the total population in period III was 8.7% (95% CI: 7.6%-9.8%). The estimated carrying capacity ‘K’ for the moult counts was 34,000 (95% CI: 25,000–43,000; Fig 2).

**Table 1:** Summary of the exponential and density-dependent models fitted to the moult count (top) for all regions combined and pup counts (bottom). Variables shown are the degrees of freedom (df), log-likelihood (loglik) and AIC.

		Exponential model			Density-dependent model		
	model	df	logLik	AIC	Df	logLik	AIC
**Moult count**	year	2	-345.00	690.00	3	-345.00	696.01
	year + period	4	-322.37	654.73	5	-322.37	654.73
	year: period	4	-322.51	655.01	5	-348.62	707.23
	year * period	6	-302.39	618.83	7	-297.39	608.79
**Pup count**	year	2	-289.47	584.95	3	-289.47	584.95
	year + period	4	-277.62	565.24	5	-277.62	565.24
	year: period	4	-277.72	565.43	5	-289.62	589.24
	year * period	6	-258.48	530.96	7	-257.61	529.22

**Table 2:** Estimated average growth rates ((λ-1)x 100) in the moult counts (top) and pup counts (bottom) for the different regions of the Wadden Sea and periods of the study, based on the best fitting exponential model (year * period * region). Regions: the Netherlands (NL), Lower Saxony (LS), Schleswig-Holstein (SH) and Denmark (DK). Periods: 1974–1987 (I), 1989–2001 (II), 2003–2014 (III).

		Period		
**moult **		**I**	**II**	**III**
Region	**NL**	5.4% (4.1,6.8)	17.9% (16.3,19.5)	10.6% (9,12.3)
	**LS**	6.7% (5.4,8)	12.4% (10.9,13.9)	8.5% (6.9,10.2)
	**SH**	6.7% (5.5,8.0)	12.8% (11.3,14.4)	7.1% (5.6,8.7)
	**DK**	12.3% (10.9,13.8)	8.6% (7.1,10.0)	9.9% (8.3,11.6)
	**Wadden Sea**	7.2% (6.4,8.1)	12.7% (11.7,13.8)	8.7% (7.6,9.8)
**pups **		**I**	**II**	**III**
Region	**NL**	5.7% (3.4,8)	17.7% (15.3,20.1)	10.7% (8.4,13.0)
	**LS**	5.4% (3.5,7.3)	11.3% (9.2,13.4)	7.9% (5.7,10.1)
	**SH**	5.6% (3.8,7.4)	16.6% (14.4,18.8)	8.8% (6.6,11.0)
	**DK**	12.7% (10.4,15.0)	9.7% (7.5,11.9)	8.5% (6.2,10.9)
	**Wadden Sea**	6.3% (5,7.5)	14.1% (12.7,15.6)	8.9% (7.4,10.5)

Using a correction factor of 68% [58], based on the average proportion of the seals hauled out in august, the estimated total harbour seal population size grew during the whole study period (1974–2014) from approximately 4,500 animals to 39,000 animals.

The maximum pup numbers in the Wadden Sea grew from 687 in 1974 to 8561 in 2014 and trended in a similar pattern to the moult counts (Fig 2 and Table 2). The estimated drop in pup numbers as a result of the PDV epidemics seemed lower than the moult counts. In 1988, modelled pup numbers dropped 53% or 51%, respectively for the density-dependent model and the exponential model, and in 2002, modelled pup numbers dropped 39% and 37%, for the respective models.

For the moult data of the total population, the exponential population model (i.e. GLMs) that fitted best (i.e. lowest AIC) was one where both the height and growth rate differed between periods (i.e. model year * period, Table 1). Adding this interaction led to a substantial improvement in the model fit (i.e. higher log-likelihood). For the same data the density-dependent model led to a drop in the AIC from 619 (i.e. exponential model) to 609, suggesting that the growth rate in the total population could have levelled off. For the pup data, the density-dependent model only led to a minor improvement (AIC declines from 531 to 529), and hence there was limited support for a slowing down of the growth rate in pup production.

### Regional differences

**Fig 3:**
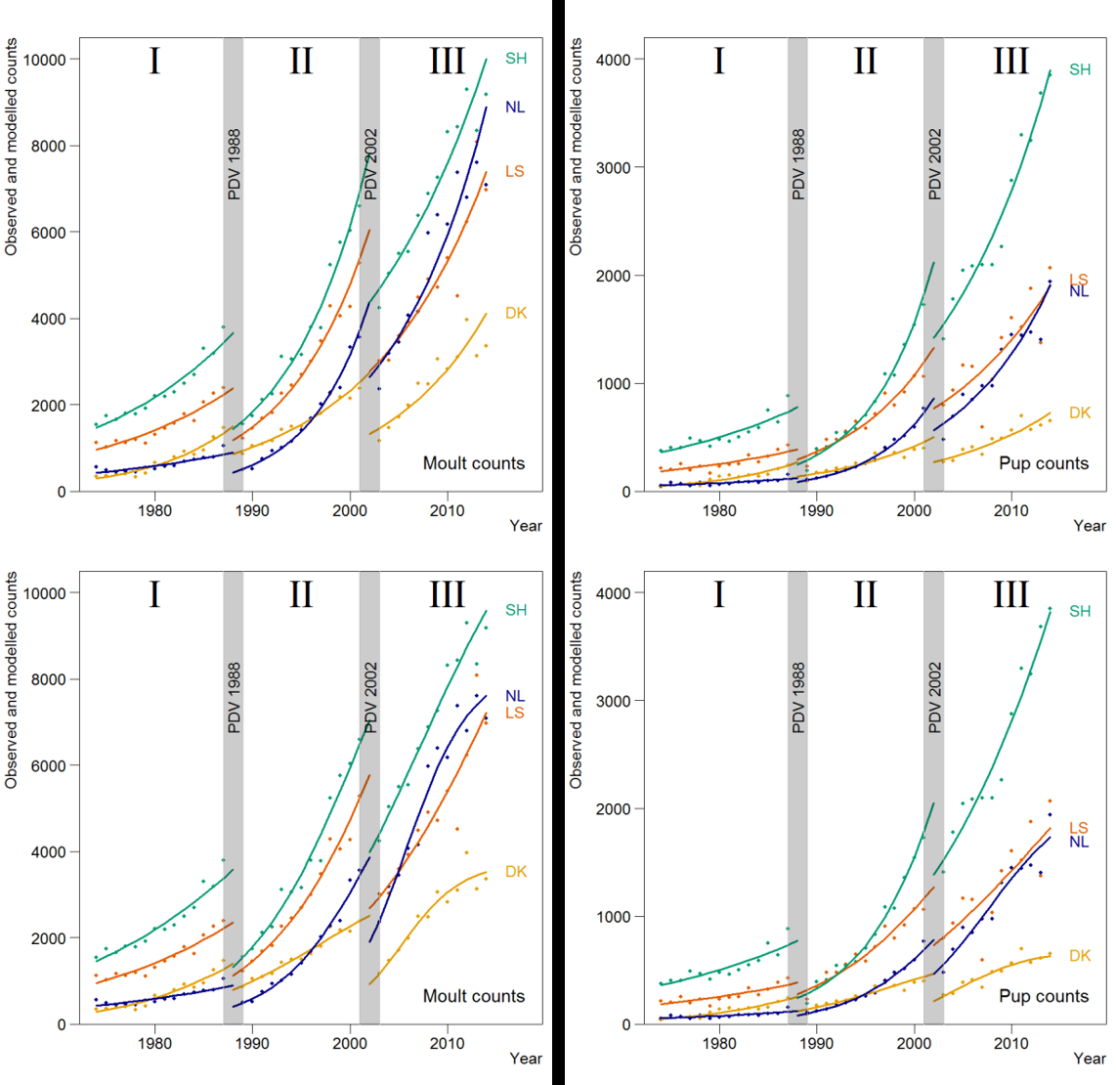
Results for the best fitting exponential (top) and density dependent models (bottom) for the harbour seals in the four regions (the Netherlands (NL), Lower Saxony (LS), Schleswig-Holstein (SH) and Denmark (DK)) of the Wadden Sea. The lines represent the estimates for the moult counts (left) and pup counts (right), and dots the observed maximum counts.

In many ways, the developments in the pup counts were similar to the moult counts (Fig 3). For both counts, a model where both the number of seals and growth rate differed between periods and regions (i.e. model year * period* region; Table 3) was the best model.

**Table 3:** Summary of both the exponential and density-dependent model fitted to the moult count (top) and pup counts (bottom). Variables shown are the degrees of freedom (df), log-likelihood (logLik) and AIC.

		Exponential model			Density-dependent model		
	Model	df	logLik	AIC	df	logLik	AIC
**Moult count**	Year*period+region	9	-1181.58	2383.16	13	-1207.01	2440.03
	year*period+region *period	15	-1093.56	2219.13	19	-1072.88	2183.77
	year*period*region	24	-1043.95	2137.89	28	-1026.71	2109.43
**Pup count**	Year*period+region	9	-966.03	1952.07	13	-972.32	1970.64
	year*period+region *period	15	-884.30	1800.61	19	-872.27	1782.54
	year*period*region	24	-855.75	1761.50	28	-850.3	1756.61

All regions showed a general recovery, interrupted in 1988 and 2002 by the PDV-epizootic events. However, the speed of recovery varied between the regions (Fig 3). Throughout the years, the highest moult and pup counts occurred in Schleswig-Holstein. The only exception was in the years just after the 1988-epizootic, where the PDV caused a drop of 69% in pup numbers of Schleswig-Holstein, while in Lower Saxony and the Netherlands, the pup production was only reduced by 27% and 31%, respectively (Table 4). Interestingly, during period II, the pup numbers in Schleswig-Holstein recovered, and the area was re-affirmed as the stronghold for pup production by the population. In the 1970’s, just after hunting ceased, Denmark and the Netherlands were the regions with the lowest numbers, but after the first epizootic, the number of animals observed in the Netherlands grew most, while the growth in Denmark seemed to level off, especially following the second, 2002-epizootic event.

**Table 4:** Estimated mortality (in the moult counts), or reduction of the pup production during PDV in 1988 and 2002 based on density dependent model (top) and exponential model (bottom).

		PDV 1988		PDV 2002	
Density-dependent		moult	pup	moult	pup
**Region**	**NL**	55%	31%	51%	40%
	**LS**	52%	27%	53%	42%
	**SH**	63%	69%	43%	32%
	**DK**	43%	53%	63%	54%
** **	**Wadden Sea**	56%	53%	50%	39%
**Exponential**					
**Region**	**NL**	51%	27%	40%	34%
	**LS**	51%	24%	54%	42%
	**SH**	61%	68%	44%	33%
	**DK**	42%	49%	52%	46%
	**Wadden Sea**	54%	51%	47%	37%

As pup numbers in the Netherlands grew faster than the Wadden Sea average, numbers in this area outgrew Denmark and, in the course of the study period, approached the numbers in Lower Saxony. Compared to other regions, the numbers in Denmark grew less and were more affected by the second PDV epizootic. At the end of period II, fewer pups were born in the Danish Wadden Sea, compared to the Netherlands and, by 2014, pup numbers in Denmark represented less than 10% of the total pup production.

**Fig 4:**
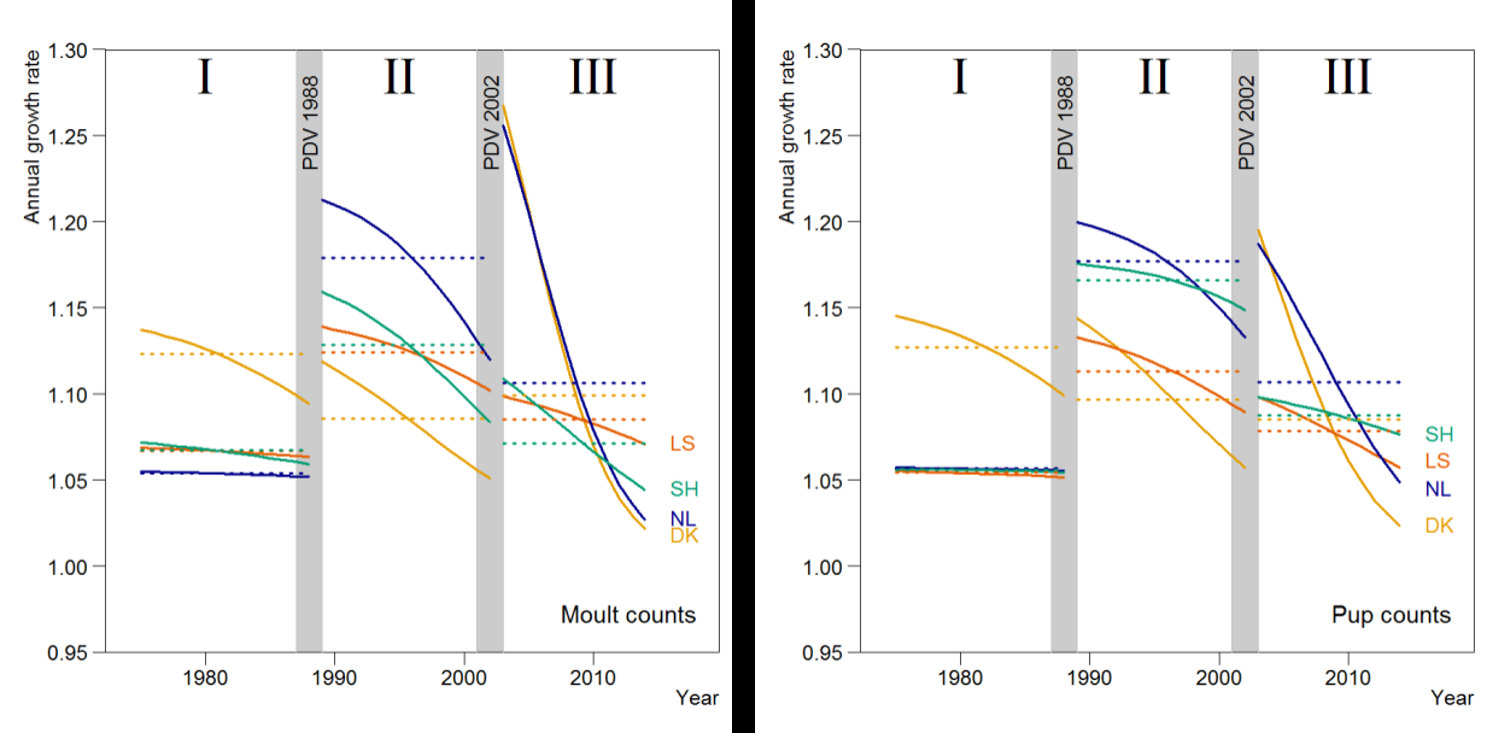
Density-dependent annual growth rates estimates for the different regions of the Wadden Sea, 1974–2014 based on the harbour seal moult counts (left), and the pup counts (right). Dotted lines show the growth rates of the best fitting exponential model, as a comparison.

Estimated average growth rates are summarised in Table 2 and shown in Figs 3 and 4. The better fit of the density-dependent models indicated a possible slowing down in growth during the study period. This was most obvious in Denmark where, in the period after the first epidemic in 1989–2002, the growth remained lower than in the other regions, while the total Wadden Sea population was growing close to its assumed intrinsic rate of increase (13%) [57]. In the last period, growth rates in all regions had dropped.

In the first period (I) from 1974 up to the PDV epidemic of 1988, the growth rate in both the moult and pup counts in Denmark was by far the largest (Table 2 and Fig 4). After the first epidemic in 1988 (period II), the highest growth rate was observed in the Netherlands, while growth in the German regions approximated the intrinsic growth rate estimated for this species, and growth in Denmark slowed down. For all regions, the density-dependent model showed an initial high rate after the epizootic, which slowed down gradually.

Overall the growth in pup numbers was similar to the growth rate in moult counts. However, there were some differences (Figs 3 and 4). For example, between 1989 and 2002 in Schleswig-Holstein and initially also in Denmark and The Netherlands, there was a substantially higher growth rate in pup number compared to the growth in moult counts.

**Fig 5:**
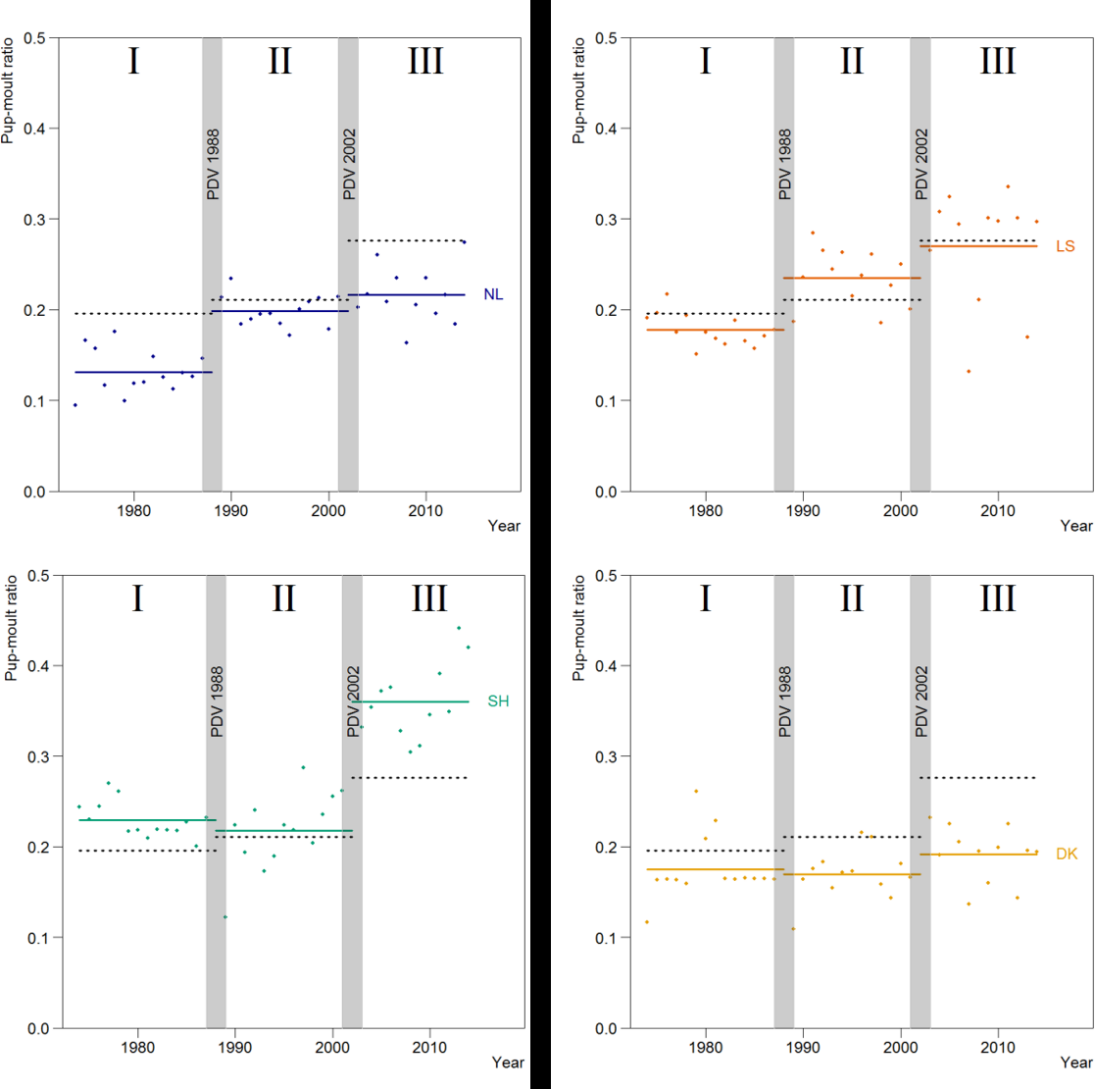
Observed pup numbers as a proportion of total seal observed during the moult in different regions of the Wadden Sea and the three time periods (I, II and III). Within each period, solid coloured lines indicate average model estimates for the region, and black dotted lines indicate the average for the Wadden Sea.

The proportion of pups (pup/moult count) for the different regions changed over time (Fig 5). Overall the largest proportion of pups was observed in the third period. Schleswig-Holstein consistently had the highest proportion of pups. Generally, the proportions of pups were lower in the Dutch and Danish regions, than in the German regions.

**Fig 6:**
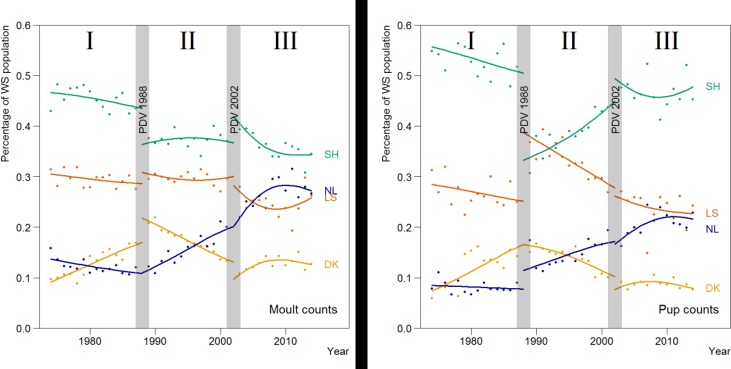
Observed and estimated proportion of harbour seals in the different regions of the Wadden Sea (green = Schleswig-Holstein, red = Lower Saxony, blue = Netherlands and yellow = Denmark) expressed as percentage of the total population, based on counts during the moult period (A) and numbers of pups (B).

The relative importance of the different regions from a population perspective changed over time. Schleswig-Holstein remained the strong-hold of the population, with 35 to 45% of the moulting seals and 35 to 55% of the pups (Fig 6). Interestingly, the sharp drop in the number of pups born in Schleswig-Holstein just after the first PDV in 1988, recovered during the following period. The opposite happened in Lower Saxony, where the relative number of pups counted increased from 27% to 40% during the 1988 PDV event, but then steadily declined during the following period II. Most growth over time was in the Netherlands, with approximately 10% of seals and 10% of pups present in the first period, rising to 25% of seals and 20% of pups in the third period.

## Discussion

The recovery of the Wadden Sea harbour seal population has been an ongoing process since the hunting ceased progressively in the different regions between 1962 and 1976 [32, 38, 39, 41, 57, 59–61]. Clearly, the increase was slowed down by the occurrence of two PDV epizootics in 1988 and 2002 killing on both occasions around over 50% of the population [38, 39, 57]. Nevertheless, the population recuperated after the epizootics and continued to grow. The number of moulting seals grew almost tenfold throughout the forty-year study period (1974–2014). Growth rates measured in this study differ only slightly from earlier studies of the Wadden Sea harbour seal population [38, 58].

For the pups, with clear peak timing in birth, the maximum number is likely to be the best estimate and index for the pup production. Despite the shifted forwards of the peak in the course of the years [62, 63]. During the moult however, numbers of animals hauled out on the sandbanks show a less clear peak. This is because they represent the sum of different age classes that haul-out in different proportions in relation to their specific moult timing [53]. Possibly, if the moult counts were averaged, this would buffer the potential effects on the variance introduced by tide, weather and occasional disturbances, however, this was not possible in this study as only the maximum count was saved in the database for some of the early years.

As the hunting ban was implemented gradually throughout the Wadden Sea, it is to be expected that initially, the dynamics of the recovery were different for the different regions, depending on the timing of the ban. For example, in 1974, the Netherlands hunting had been banned for more than a decade; the German regions had just banned hunting while in Denmark hunting continued until 1976. One could expect the changes in the first period to mirror this. In contrary, exponential growth rates are highest in Denmark and lowest in the Netherlands, where high pollutant (PCB) burdens affected the reproduction [3, 28, 38].

Throughout the study period, pup production was relatively low in the Netherlands (on average 18% of all Wadden Sea pups were born there), compared to the moult growth, which was 17.9% and 10.6% respectively for period II and III). Especially in the latest period between 2002 and 2014 Schleswig-Holstein and Lower Saxony performed at or above the Wadden Sea average, with average pup ratios of almost 35% and 28% respectively, while Denmark and the Netherlands show a pup ratio below average, respectively 19% and 21%. Even though by far most pups were born in Schleswig-Holstein (46% of all pups), in absolute numbers, but also in relative numbers, moult growth rates there were not higher, but close to the average of the Wadden Sea, (12.7% in period II and 8.7% in period III). To a lesser extent this also held for Lower Saxony (26% of the pups). It is therefore likely that there was a net influx from other regions into the Netherlands during moult, especially between the epizootic events, when growth rate in the Netherlands (17.9%) was well above the maximum intrinsic rate of increase estimated at 13% [57].

The fact that growth rates in pup numbers in Schleswig-Holstein were significantly higher than the growth in moult counts indicates that in Schleswig-Holstein, the number of breeding females (producing a pup) grew at a higher rate than the numbers during the moult. This implicates that compared to other areas, a large proportion of the seals in Schleswig Holstein migrate out of the region after the breeding season (these could be females, but also males or juveniles).

Extreme high growth rates in the moult count found just after the epizootics (Fig 4) and high growth in pup numbers indicate a change in demographic structure throughout the Wadden Sea, as was observed in the Kattegat-Skagerrak [57]. In periods II and III, in the absence of hunting and as pollution diminished, circumstances in the regions should have been more similar, however regional differences in growth rates persisted, albeit becoming less obvious.

As throughout the study the growth rate of the total Wadden Sea population did not exceed the intrinsic rate of increase for the species, it seems unlikely that the growth was influenced, let alone fuelled by immigration from colonies outside the Wadden Sea. This is supported by earlier findings that indicate the Wadden Sea harbour seal population being a distinct genetic population [34, 35] though recent findings indicated there was a strong connection with harbour seals from France and southern UK [64]. In addition to this, the occurrence of virus epizootics did affect population growth temporarily, but did not prevent the population from continuing its recovery. Interestingly, as the model including both the different periods and the regions shows the best fit, there seems to be significant differences between the recoveries in the different regions, which on its turn are affected by the PDV outbreaks.

The density-dependent model performs slightly better than the exponential model (Table 3), indicating, though not conclusively, that the population growth might be affected by the limits of the carrying capacity of the area. However, biased estimates of the rates of increase in the population can be expected, as a result of age specific mortality, for example during the PDV epidemic, due to variation in haul-out between the different age and sex classes, especially in the five years following the epidemic [53, 57, 65]. To test a possible effect the density-dependent model was also fitted to the count data excluding the first 5 years following the PDV epidemics. When excluding the first five years after the two epidemics, the difference in AIC between the density-dependent (AIC: 1600.46 for moult counts) and exponential models (AIC: 1602.14) are much less prominent, providing less support for a hypothesis that the population is approaching its carrying capacity. However for this latter exercise, much less points were available, and therefore the ability to detect a density dependent effect is reduced.

In the third period after the 2002 epizootics, average growth rate was positive in the total Wadden Sea (8.7% pa), but was below the intrinsic rate across the regions. Based on the AIC, the density dependent model performs slightly better than an exponential model, again indicating a possible start of density dependence. The density-dependent model indicates that the estimated carrying capacity *K* would currently be at a population size of 50,000 (95% CI 37,000–63,000) animals when correcting for animals not seen during the counts (correction factor 1.47 [58]). The population estimate for 2014, using the same factor is almost 35,000 animals. In future years it should become clear whether the population is reaching the carrying capacity for the current Wadden Sea ecosystem.

### Regional differences in population development

The potential reasons for the observed differences in growth rates between regions include human related effects such as disturbance, pollution and management but also the effects of the PDV epizootics and environmental differences such as area size (Table 5).

**Table 5:** Overview of differences between regions in the Wadden Sea, including area sizes and seal densities at the beginning and end of the study period, timing of stranding expressed as percentage of the total number of animals found dead during the PDV epizootics of 1988 and 2002. Adopted after [40], hunting regulations and rehabilitation.

		Netherlands	Lower Saxony	Schleswig-Holstein	Denmark	Total Wadden Sea
**PDV**						
25% found dead	1988	8-Aug	8-Aug	27-Jul	5-Jul	
50% found dead	1988	4-Sep	4-Sep	17-Aug	3-Aug	
25% found dead	2002	21-Aug	3-Sept	10-Sept	9-Sept
50% found dead	2002	2-Sep	18-Sep	21-Sep	20-Sep	
**Area size/ density**						
Length North Sea coastline (km)		158	161	101	80	500
*Seal density moult/pups(km*)	1974	3.48/ 0.34	7.00/ 1.34	15.29/ 3.73	4.38/ 0.51	7.14/ 1.37
	2014	*39*.*65/12*.*29*	*42*.*62/12*.*84*	*74*.*06/38*.*15*	*38*.*95/ 8*.*18*	*47*.*44/17*.*03*
Wadden Sea area (km2)		2685	2462	2534	706	8387
*Seal density moult/pups(km2)*	1974	0.20/ 0.02	0.46/ 0.09	0.61/ 0.15	0.50/ 0.06	0.43/ 0.08
	2014	*2*.*33/ 0*.*72*	*2*.*79/ 0*.*84*	*2*.*95/ 1*.*52*	*4*.*41/ 0*.*93*	*2*.*83/ 1*.*02*
Subtidal area		53%	39%	44%	46%	45%
**Hunting**						
Until 1900		Open hunt	Open hunt	Open hunt	Open hunt	
From 1934		Open hunt	Hunt regulated	Hunt regulated	Open hunt	
Hunting ban	since	1962	1971	1973	1977	
**Rehabilitation centres**	since	1952* 1971	1978	1985	1978–1995**	

* In the Netherlands there were two rehabilitation centres.

** In Denmark rehabilitation ceased after 1995.

The trilateral agreement has insured a similar management of the Wadden Sea area, with regards to seals. This includes for example disturbance of the seals, but excludes effects extending from the adjacent North Sea. The southern North Sea area bordering the Wadden Sea is one of the busiest marine areas in the world with intensive fishing activities and shipping to and from large harbours, such as Rotterdam, Hamburg and Antwerp. In addition there has been an extensive growth in exploitation of fossil fuels: comprising seismic surveys, platform construction, pipe-laying and drilling, growing areas of sand mining and recent development of wind farms in the Economic Exclusive Zones of all the Wadden Sea countries. However, though these activities might affect the carrying capacity of the area, there is no indication that one region has been consistently more affected by these activities than the others, in such a way that might drive the differences found. More likely, other factors have played a role in the differences found between the regions.

PCB’s were found to cause reproductive failure [28] in the 1970’s, especially in the Netherlands. Levels in seals from the Dutch Wadden Sea were ten times higher than in Denmark and Schleswig Holstein [28]. The latter regions showed the highest proportion of pups through the period, possibly supporting this hypothesis. However, as many pollutants were banned, the situation ameliorated and gradually the differences in levels of PCB in the Wadden Sea have become marginal [38, 66, 67]. Between the two epizootic events (1989–2001, period II), average growth rate of the whole population attained its highest level (12.7% pa), matching the intrinsic rate of increase for this seal species [57]. This could indicate that the earlier problems in relation to pollution had become of minor importance. Possibly, the PDV epidemics could have selectively eliminated many animals carrying a high pollutant burden and hampered in their reproduction, as a result reproduction was somewhat normalised after the first outbreak and a high growth could be attained [38].

Our study shows marked differences between the regions when looking at the mortality during the PDV outbreaks (Table 4). It still remains unclear, however, how the two occurrences of PDV in 1988 and 2002 might have been responsible for the observed patterns in the seal populations’ recovery. Though both occurrences started on the island of Anholt, east of Denmark in the Kattegat, the timing and spread were different [68]. In 1988 the virus swept through the Wadden Sea from east to west, while practically the contrary was the case in 2002 as a second epicentre seemed to have started in the Netherlands just after the first outbreak at Anholt. Moreover, for most regions the virus was several weeks later in 2002. Especially for Schleswig-Holstein and Denmark, the virus outbreak came later to the Wadden Sea, possibly affecting other age or sex groups of the population than in 1988, as the haul-out patterns of the different groups are expected to change throughout the breeding and moulting season. However, these differences do not seem to explain the observed differences in mortality of the two occurrences. Despite the later arrival of PDV in Lower Saxony, total mortality was similar between the two epizootics and to a lesser extent this was also the case in the Netherlands. In both regions pup mortality was higher than in 1988. On the other hand, despite a two month difference, the mortality in Denmark was much higher in 2002, and with a similar timing for both epizootics, mortality in Schleswig-Holstein was much lower (Table 4).

The population is currently well below critical herd immunity for PDV, which caused a much higher mortality in the earlier epizootics [69]. Though the details of the start of a new outbreak are not understood, it is advisable consider a reoccurrence in the near future, and keep an adequate monitoring operable. This is also the case for other diseases. In the autumn of 2014 an avian flu epidemic caused elevated mortality in the eastern Wadden Sea area (Denmark and Schleswig-Holstein) and practically no effect in the west (Lower Saxony and the Netherlands) [70]. This event occurred after our study period.

The carrying capacity for the number of animals hauled out within regions may be influenced by size or quality of the habitats available. This could be either feeding habitats or habitats for resting and breeding. Telemetry data show that, even though they haul-out in the Wadden Sea, the majority of the seals forage in the adjacent North Sea [71–75]. Within the Wadden sea area the regions vary considerably in size (Table 5). Of the four Wadden Sea regions, Denmark is the smallest both in coastline (a proxy for accessibility to feeding grounds) and area (possible haul-out), and is therefore expected to have the lowest carrying capacity. Possibly, the higher seal density per km^2^, as a result, might explain the slower growth observed in the later periods, although the area does not hold the highest density of pups, nor the most animals per km coastline. However the relation between seal density and surface area or coast length is not always clear when observing the densities in both the numbers of seals and pups born. For example, based on coastline, Schleswig-Holstein clearly is more densely used than are the other regions and especially for pup production, which in Schleswig-Holstein is 2–4 times the density of other regions. Interestingly, the Netherlands is the largest both in coastline and area, while numbers were initially lowest, the highest growth has been observed in this region.

Though there are differences in growth rates that are possibly related to the carrying capacity, it is unlikely that the available land habitat would be the sole driver of the differences in growth between the regions, especially in the earlier periods, when numbers were still relatively low. We conclude therefore that there must have been other factors that contributed to these differences.

Since 1952, seals have been captured, rehabilitated and released in the Wadden Sea. While in Denmark seals which are found orphaned or injured have not been taken in for rehabilitation since 1995, in the Netherlands and Germany, rehabilitation has been common practice throughout the study period. Two rescue centres have been active during the study period in the Netherlands. In Germany, there are also two rescue centres; one in Lower Saxony, and one in Schleswig-Holstein. Though total numbers of seals (adults and pups) released into the wild were relatively low, and are believed to amount to approximately two hundred seals in average and tree hundred in extreme years (to this date exact numbers on released animals have not been published) this might also have somewhat affected the observed changes in the population, especially when the total number of seals were low. In order to study the exact magnitude of the effect and differences between regions, more information is needed.

Historical findings show that seals throughout the Wadden Sea have been hunted by man for centuries, ever since man colonised the area around 3500 BC [76]. In addition to hunting for subsistence or profit, seals became persecuted because of their perceived or actual impacts on fish catches and damage to fishing gear. In the Netherlands, for example, one of the first bounty hunts was proclaimed in the late 1500’s [36, 77]. Generally, pressure increased as better hunting techniques developed–especially through modernisations in firearms which made hunting much more effective [36]. However, during the 19^th^ and 20^th^ centuries, regional differences developed as the different countries applied different management strategies. The situation in the Netherlands was very similar to Denmark, where more or less any citizen could hunt for seals. Bounty systems effectively reduced the seal population significantly [22]. Especially after the 2^nd^ World War in the Netherlands, annual hunting mortality was estimated to be 55% of the total counts [78]. In contrast, hunting mortality in Germany was estimated to be much lower, 7%. Moreover, hunting during the pupping season was forbidden from 1938 onwards [79]. Following the hunting law, only specially appointed game keepers, “Jagdaufseher”, were entitled to capture and kill seals. Seals were completely protected from hunting in the Netherlands in 1962, in Germany 1971–73 and in Denmark in 1976.

We hypothesise that the differences in hunting regulations and pressure in the first half of the 1900s, which led to local dissolution of seal breeding grounds in the Danish and Dutch regions, could be one of the most important causes for the observed differences in seal densities during the breeding period.

**Fig 7:**
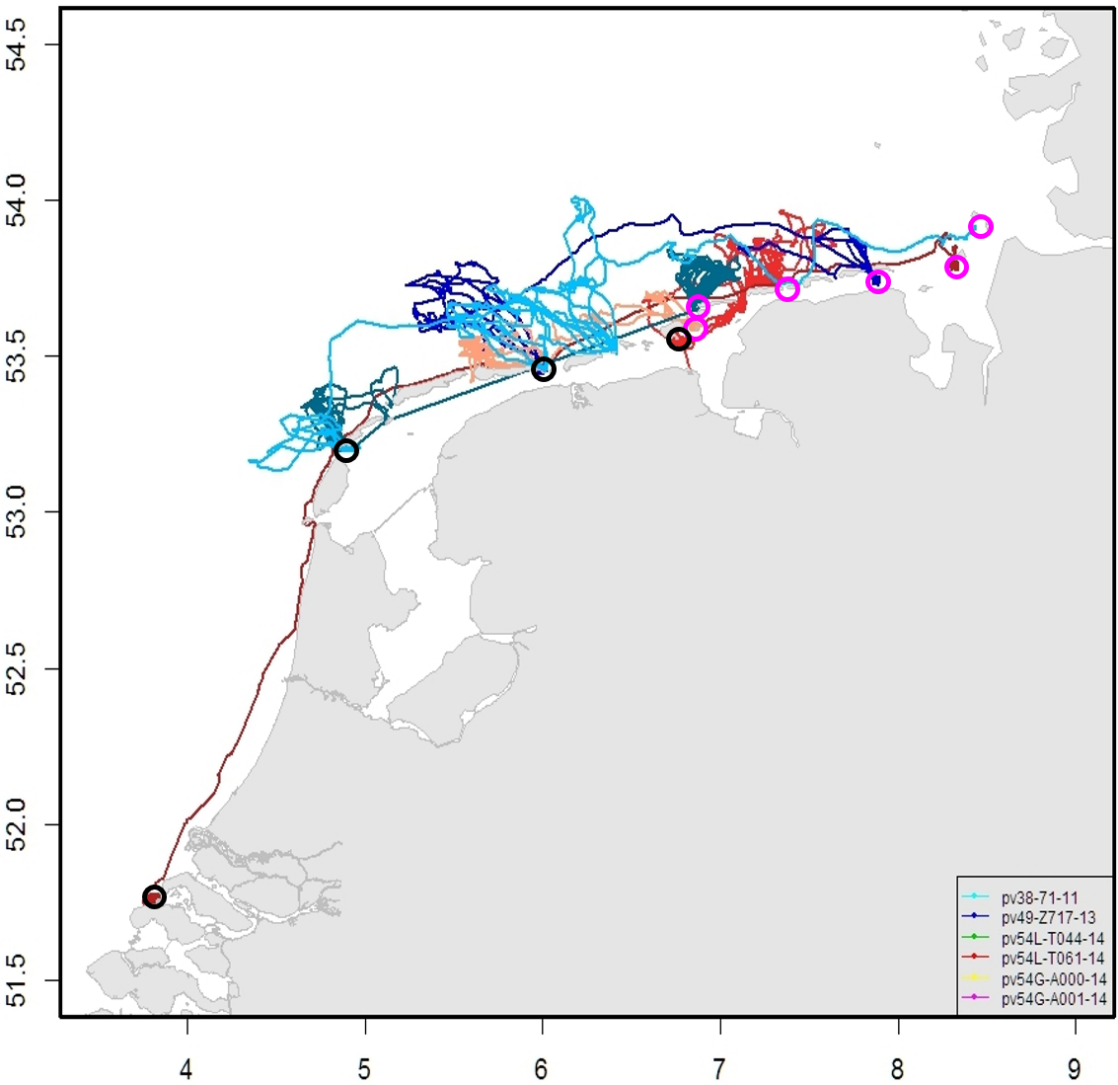
Examples of tracks of six adult females tagged in the Netherlands (black circles), migrating towards Germany during the pupping season (pink circles)[72–74].

The mechanism for sustaining the different pup densities could be the high degree of site fidelity and natal philopatry shown by harbour seals [80–83]. The assumption is that relatively many females and their pups survived in the more sustainably hunted German breeding area, as less seals were killed and mothers and pups were not hunted during breeding. During other periods seals could redistribute, only to come back to breed. As 70% of the pups are born in the German regions, there must be an unequal post-breeding dispersal of reproductive females throughout the area. As such, more pups could be born in the preferred breeding areas than can be expected from the seal distribution outside the breeding season. This breeding migration towards the German Wadden Sea has been observed from the Netherlands on several occasions ([72–74]; Fig 7). Some indication for this behaviour can also be found in the recovery of Schleswig-Holstein after the first PDV epidemic. Then pup counts dropped below those of Lower Saxony indicating that breeding animals had been killed disproportionally in that region. However within period II pup numbers grew and Schleswig-Holstein attained higher pup numbers compared to the other areas. Possibly this recovery was fuelled by animals returning to their natal sites as they reached reproductive age. Possibly this effect was magnified in the Netherlands by the suppression of the reproduction by PCB’s, especially in period I and beginning of II [28, 38].

For the Wadden Sea, the German regions could be considered to be sources and Denmark and the Netherlands sinks (Pulliam 1988). However pupping habitat quality, in terms of available sandbanks, access channels, protection from disturbance, seems to be relatively uniform throughout the region. Therefore, the regional developments are more likely driven by breeding and natal site-fidelity. A form of “hidden source sink dynamics” [84, 85], may more accurately describe the situation. Here, animals migrate from areas where more pups are born to areas where fewer pups are born, prompting growth throughout the range.

Migration between sites could also help to explain the very high growth rates attained in the Netherlands [11, 37]. Previously, an unbalanced age structure has been proposed as an explanation for this growth, where a relatively higher proportion of adult females could produce a high pup rate for a number of years [65, 80]. However, such an imbalance is unlikely to prevail for decades. In the years between the epizootic events, numbers in the Dutch Wadden Sea grew at an average of 17.9% pa, while for the total Wadden Sea; growth remained below 13% pa. The latter is close to the intrinsic rate of increase for harbour seals [57]. So more likely, high levels of migration explained the higher growth rate in the Netherlands at that time.

## Conclusion

Although the international Wadden Sea could be regarded as a single connected ecological system, where seals are capable of migrating between the geo-political regions, large regional differences within the harbour seal population growth rate and pupping success are apparent. Though there seems to be some factors differing between the regions, differences in hunting pressure and in regulations enforced in the past seem to be a dominant factor in the observed patterns.

These findings reveal that different management regimes operating 40 years ago still influence the current population structure, distribution and demography. This long-term effect is a consequence of the longevity of the animals and their site faithfulness during breeding. It is important to realise that, management decisions regarding seals, could affect the distribution and development of populations even long after their implementation. The same could hold for many other species though few populations have been studied or monitored as long as the Wadden Sea harbour seals. These effects could also occur under much less drastic management regimes, such as closure or opening of areas for the public, or for (industrial) development, affecting the carrying capacity of the area, but might also affect certain groups in the population more than others. Though the disturbances might be less crucial for the survival of individual animals than hunting, they could cause displacement which in-turn might have long term effects.

## Supporting information

S1 Table Overview of number of surveys per year. M = moult, P = pupping season.
(DOCX)

S2 Table Annual maximum counts for total numbers (during moult) and pups. Left column indicates the periods.
(DOCX)

## References

[1] Burchard I. Anthropogenic impact on the climate since man began to hunt. Palaeogeography, Palaeoclimatology, Palaeoecology. 1998;139(1):1–14.

[2] Pauly D, Christensen V, Dalsgaard J, Froese R, Torres F Jr. Fishing down marine food webs. Science. 1998;279(5352):860–3. 9452385 10.1126/science.279.5352.860

[3] Reijnders PJH. Management and conservation of the harbour seal, *Phoca vitulina*, population in the international Wadden Sea area. Biological Conservation. 1980;19(1):213–21.

[4] Woodroffe RT, Simon; Alan Rabinowitz. The impact of human-wildlife conflict on natural systems. Conservation Biology Series-Cambridge. 2005;9:1.

[5] Crain CM, Kroeker K, Halpern BS. Interactive and cumulative effects of multiple human stressors in marine systems. Ecology Letters. 2008;11(12):1304–15. doi: 10.1111/j.1461-0248.2008.01253.x 19046359 10.1111/j.1461-0248.2008.01253.x

[6] Daszak P, Cunningham AA, Hyatt AD. Emerging Infectious Diseases of Wildlife—Threats to Biodiversity and Human Health. Science. 2000;287(5452):443–9. 10642539 10.1126/science.287.5452.443

[7] Descamps S, Aars J, Fuglei E, Kovacs KM, Lydersen C, Pavlova O, et al Climate change impacts on wildlife in a High Arctic archipelago–Svalbard, Norway. Global Change Biology. 2017;23(2):490–502. doi: 10.1111/gcb.13381 27250039 10.1111/gcb.13381

[8] Munson L, Terio KA, Kock R, Mlengeya T, Roelke ME, Dubovi E, et al Climate Extremes Promote Fatal Co-Infections during Canine Distemper Epidemics in African Lions. PLOS ONE. 2008;3(6):e2545 doi: 10.1371/journal.pone.0002545 18575601 10.1371/journal.pone.0002545 PMC2435602

[9] Wolff WJ, Zijlstra JJ. Management of the Wadden Sea. Helgolander Meeresuntersuchungen. 1980;33(1–4):596–613.

[10] Caughley G, Sinclair ARE. Wildlife Ecology and Management Cambridge UK: Blackwell Science; 1994 334 p.

[11] Reijnders PJH. Management and conservation of the harbour seal, *Phoca vitulina*, Population in the International Wadden Sea Area. Biological Conservation. 1981;19(3):213–21.

[12] Halpern BS, Walbridge S, Selkoe KA, Kappel CV, Micheli F, D'Agrosa C, et al A global map of human impact on marine ecosystems. Science. 2008;319(5865):948–52. doi: 10.1126/science.1149345 18276889 10.1126/science.1149345

[13] Reijnders P, Brasseur SMJM, van der Toorn J, van der Wolf P, Boyd I, Harwood J, et al Seals, Fur Seals, Sea Lions and Walrus, Status survey and conservation action plan: USA: Kelvyn Press; 1993.

[14] Burkey TV. Extinction in nature reserves: The effect of fragmentation and the importance of migration between reserve fragments. Oikos. 1989;55(1):75–81.

[15] Clapham PJ, Aguilar A, Hatch LT. Determining spatial and temporal scales for management: lessons from whaling. Marine Mammal Science. 2008;24(1):183–201.

[16] Andersen L, Olsen MT. Distribution and population structure of North Atlantic harbour seals (*Phoca vitulina*). NAMMCO Scientific Publications, 8 2010 p. 15–35.

[17] Kokko H, Helle E, Lindstrom J, Ranta E, Sipila T, Courchamp F. Backcasting population sizes of ringed and grey seals in the Baltic and Lake Saimaa during the 20th century. Annales Zoologici Fennici. 1999;36(2):65–73.

[18] Härkönen T, Harding KC, Goodman SJ, Johannesson K. Colonization history of the baltic harbor seals: Integrating archaeological, behavioral, and genetic data. Marine Mammal Science. 2005;21(4):695–716.

[19] Harding KC, Härkönen TJ. Development in the Baltic grey seal (*Halichoerus grypus*) and ringed seal (*Phoca hispida*) populations during the 20th century. Ambio. 1999;28(7):619–27.

[20] Brasseur SMJM, van Polanen Petel TD, Gerrodette T, Meesters EHWG, Reijnders PJH, Aarts G. Rapid recovery of Dutch grey seal colonies fueled by immigration. Marine Mammal Science. 2015;31(2):405–26.

[21] Patterson TA, Sharples RJ, Raymond B, Welsford DC, Andrews-Goff V, Lea MA, et al Foraging distribution overlap and marine reserve usage amongst sub-Antarctic predators inferred from a multi-species satellite tagging experiment. Ecological Indicators. 2016;70:531–44.

[22] Joensen AH, Søndergaard NO, Hansen EB. Occurrence of seals and seal hunting in Denmark. Dan Rev Game Biol 1976;10:1–20.

[23] Reijnders PJH. Retrospective population analysis and related future management perspectives for the harbour seal *Phoca vitulina* in the Wadden Sea. Netherlands Institute for Sea Research, Publication Series. 1992;20:193–7.

[24] Vooys, Brasseur SMJM, Meer, Reijnders. Analyses of four centuries of bounty hunting on seals in Zeeland, SW-Netherlands. Lutra. 2012;55(1):55–65.

[25] Drescher HE, Harms U, Huschenbeth E. Organochlorines and heavy metals in the harbour seal *Phoca vitulina* from the German North Sea Coast. Marine Biology. 1977;41(1):99–106.

[26] Brouwer A, Reijnders PJH, Koeman JH. Polychlorinated Biphenyl (Pcb)-contaminated fish induces vitamin-a and thyroid-hormone deficiency in the common seal (*Phoca vitulina*). Aquat Toxicol. 1989;15(1):99–105.

[27] Reijnders PJH. On the extinction of the Southern Dutch harbour seal population. Biological Conservation. 1985;31(1):75–84.

[28] Reijnders PJH. Reproductive failure in common seals feeding on fish from polluted coastal waters. Nature. 1986;324(6096):456–7. doi: 10.1038/324456a0 3785423 10.1038/324456a0

[29] Lonergan M, Duck CD, Thompson D, Mackey BL, Cunningham L, Boyd IL. Using sparse survey data to investigate the declining abundance of British harbour seals. Journal of Zoology. 2007;271(3):261–9.

[30] SCOS. Scientific advice on matters related to the management of seal populations: 2010. Available from: http://www.smru.st-and.ac.uk/documents/341.pdf.

[31] SCOS Scientific advice on matters related to the management of seal populations: 2015. Available from: http://www.smru.st-and.ac.uk/documents/341.pdf.

[32] Reijnders PJ, Brasseur SM, Tougaard S, Seibert U, Borchardt T, Stede M. Population development and status of harbour seals (*Phoca vitulina*) in the Wadden Sea. NAMMCO Scientific Publications. 8 2010 p. 95–105.

[33] Olsen MT, Andersen SM, Teilmann J, Dietz R, Edrén SMC, Linnet A, et al Status of the harbour seal (*Phoca vitulina*) in Southern Scandinavia In: Geneviève Desportes AB, Rosing-Asvid Aqqaluand Waring Gordon T, editor. Harbour seals in the North Atlantic and the Baltic 8 2010-09-01 ed: NAMMCO Scientific Publications; 2010.

[34] Goodman SJ. Patterns of extensive genetic differentiation and variation among European harbor seals (*Phoca vitulina vitulina*) revealed using microsatellite DNA polymorphisms. Molecular Biology and Evolution. 1998;15(2):104–18. 9491609 10.1093/oxfordjournals.molbev.a025907

[35] Stanley HF, Casey S, Carnahan JM, Goodman S, Harwood J, Wayne RK. Worldwide patterns of mitochondrial DNA differentiation in the harbor seal (*Phoca vitulina*). Mol Biol Evol. 1996;13(2):368–82. 8587502 10.1093/oxfordjournals.molbev.a025596

[36] de Vooys KGN, Brasseur SMJM, Meer Jvd, Reijnders PJH. Analyses of four centuries of bounty hunting on seals in Zeeland, SW-Netherlands. Lutra. 2012;55(5):55–65.

[37] Reijnders PJH. The effect of seal hunting in Germany on the further existence of a harbour seal population in the dutch Wadden Sea. Zeitschrift Fur Saugetierkunde-International Journal of Mammalian Biology 1983;48(1):50–4.

[38] Reijnders PJH, Ries EH, Tougaard S, Nørgaard N, Heidemann G, Schwarz J, et al Population development of harbour seals *Phoca vitulina* in the Wadden Sea after the 1988 virus epizootic. Journal of Sea Research. 1997;38(1–2):161–8.

[39] Harding KC, Härkönen T, Caswell H. The 2002 European seal plague: epidemiology and population consequences. Ecology Letters. 2002;5(6):727–32.

[40] Härkönen L, Dietz R, Reijnders PJH, Teilmann J, Harding K, Hall A, et al A review of the 1988 and 2002 phocine distemper virus epidemics in European harbour seals. Diseases of Aquatic Organisms. 2006;68(2):115–30. doi: 10.3354/dao068115 16532603 10.3354/dao068115

[41] Galatius A, Brasseur SMJM, Czeck R, Jensen LF, Armin J, Körber P, et al. Trilateral Seal Expert Group (TSEG). Aerial surveys of Harbour Seals in the Wadden Sea in 2015. Moderate impact of the 2014 influenza epidemic2015; 2016. Available from: http://www.waddensea-secretariat.org/sites/default/files/downloads/tmap/MarineMammals/harbour_seal_report_2015.pdf.

[42] Anonymous. Convention on the Conservation of Migratory Species of Wild Animals http://www.cms.int/sites/1983

[43] Reijnders PJH, Verriopoulos G, Brasseur SMJM. Status of pinnipeds relevant to the European Union Wageningen: IBN-DLO; 1997.

[44] Reijnders P, Abt K, Brasseur S, Tougaard S, Siebert U, Vareschi E. Sense and sensibility in evaluating aerial counts of harbour seals in the Wadden Sea. Wadden Sea Newsletter. 2003;28(1):9–12.

[45] Reijnders PJH, van Dijk J, Kuiper D. Recolonization of the dutch Wadden Sea by the grey seal *Halichoerus grypus*. Biological Conservation. 1995;71(3):231–5.

[46] Brasseur SMJM, van Polanen Petel TD, Gerrodette T, Meesters EHWG, Reijnders PJH, Aarts G. Rapid recovery of Dutch gray seal colonies fueled by immigration. Marine Mammal Science. 2015;31(2):405–26.

[47] Abt K, Engler J. Rapid increase of the grey seal (*Halichoerus grypus*) breeding stock at Helgoland. Helgoland Marine Research. 2009;63(2):177–80.

[48] Brasseur SMJM, Reijnders PJH, Borchardt T, Siebert U, Stede M, Ramdohr S, et al. Trilateral Seal Expert Group (TSEG) Aerial surveys of harbour seals in the Wadden Sea in 2008: Back to pre-epizootic level, and still Growing: Wadden Sea harbour seal population in 2008. Annual reports http://wwwwaddensea-secretariatorg/news/news/Seals/Annual-reports/seals2008html [Internet]. 2008.

[49] Reijnders PJH. Recruitment in the harbour seal (*Phoca vitulina*) population in the Dutch Wadden Sea. Netherlands Journal of Sea Research. 1978;12(2):164–79.

[50] Fransz H, Reijnders P. Estimation of birth rate and juvenile mortality from observed numbers of juveniles in a seal population with normally dispersed reproduction ICES CM 1978/N:7: International Council for the Exploration of the Sea; 1978.

[51] Thompson PM, Wheeler H. Photo-ID-based estimates of reproductive patterns in female harbor seals. Marine Mammal Science. 2008;24(1):138–46.

[52] Thompson PM, Harwood J. Methods for estimating the population Size of common seals, *Phoca vitulina*. Journal of Applied Ecology. 1990;27(3):924–38.

[53] Härkönen T, Harding KC, Lunneryd SG. Age- and sex-specific behaviour in harbour seals *Phoca vitulina* leads to biased estimates of vital population parameters. Journal of Applied Ecology. 1999;36(5):825–41.

[54] Turner H, Firth D. Generalized nonlinear models in R: An overview of the gnm package2015. Available from: http://CRAN.R-project.org/package = gnm.

[55] Burnham KP, Anderson DR. Model selection and multimodel inference: a practical information-theoretic approach. New York: Springer-Verlag; 2002.

[56] R Development Core Team. R: A language and environment for statistical computing Vienna, Austria: R Foundation for Statistical Computing; 2009 Available from: http://www.R-project.org.

[57] Härkönen T, Harding KC, Heide-Jørgensen MP. Rates of increase in age-structured populations: a lesson from the European harbour seals. Canadian Journal of Zoology-Revue Canadienne De Zoologie. 2002;80(9):1498–510.

[58] Ries EH, Hiby LR, Reijnders PJH. Maximum likelihood population size estimation of harbour seals in the Dutch Wadden Sea based on a mark-recapture experiment. Journal of Applied Ecology. 1998;35(2):332–9.

[59] Reijnders PJH. The harbour seal (*Phoca vitulina*) population in the Dutch Wadden Sea: Size and composition. Netherlands Journal of Sea Research. 1976;10(2):223–35.

[60] Reijnders PJH, Ries, Brasseur On the status of harbour seals in the Wadden Sea in 1995. Wadden Sea News 1996. 1996;1:31–2.

[61] Reijnders PJ, Brasseur S, Abt K, Siebert U, Stede M, Tougaard S. The harbour seal population in the Wadden Sea as revealed by the aerial surveys in 2003. Wadden Sea Newsletter. 2003;2:11–2.

[62] Reijnders PJH, Brasseur, Meesters. Earlier pupping in harbour seals, *Phoca vitulina*. Biology letters 2010;6(6):854–7. doi: 10.1098/rsbl.2010.0468 20591851 10.1098/rsbl.2010.0468 PMC3001384

[63] Cordes LS, Thompson PM. Variation in breeding phenology provides insights into drivers of long-term population change in harbour seals. Proceedings of the Royal Society B: Biological Sciences. 2013;280(1764):20130847. 10.1098/rspb.2013.0847 PMC3712417 23782881

[64] Olsen MT, Islas V, Graves JA, Onoufriou A, Vincent C, Brasseur S, et al Genetic population structure of harbour seals in the United Kingdom and neighbouring waters. Aquatic Conservation: Marine and Freshwater Ecosystems. 2017:n/a-n/a.

[65] Härkönen T, Harding K, Rasmussen TD, Teilmann J, Dietz R. Age- and sex-specific mortality patterns in an emerging wildlife epidemic: the phocine distemper in European harbour seals. PLoS One. 2007;2(9):e887 doi: 10.1371/journal.pone.0000887 17849016 10.1371/journal.pone.0000887 PMC1964516

[66] Laane, Vethaak AD, Gandrass, Vorkamp K, Köhler A, Larsen MM, et al Chemical contaminants in the Wadden Sea: Sources, transport, fate and effects. Journal of Sea Research. 2013;82:10–53.

[67] Reijnders PJ, Simmonds MP. Global temporal trends of organochlorines and heavy metals in pinnipeds In: Vos GDB, Fournier, O'Shea T.J. editor. Toxicology of marine mammals. London: Taylor and Francis; 2003 p. 491–506.

[68] Härkönen T, Dietz R, Reijnders P, Teilmann J, Harding K, Hall A, et al The 1988 and 2002 phocine distemper virus epidemics in European harbour seals. Diseases of Aquatic Organisms. 2006;68(2):115–30. doi: 10.3354/dao068115 16532603 10.3354/dao068115

[69] Härkönen T, Harding KC. Predicting recurrent PDV epizootics in European harbour seals (*Phoca vitulina*). NAMMCO Scientific Publications. 2010;8:275–84.

[70] Bodewes R, Rubio García A, Brasseur SM, Sanchez Conteras GJ, van de Bildt MWG, Koopmans MPG, et al Seroprevalence of antibodies against Seal Influenza A(H10N7) virus in harbor seals and gray seals from the Netherlands. PLoS ONE. 2015;10(12):e0144899 doi: 10.1371/journal.pone.0144899 26658347 10.1371/journal.pone.0144899 PMC4684379

[71] Brasseur SMJM Aarts GM, Meesters HWG Polanen-Petel Tv, Dijkman EM Cremer JSM, et al Habitat preferences of harbour seals in the Dutch coastal area: analysis and estimate of effects of offshore wind farms. Texel: IMARES; 2010. Contract No.: Report number C137/10.

[72] Brasseur SMJM, Aarts GM, Bravo Rebolledo E, Cremer JSM, Fey-Hofstede FE, Geelhoed SCV, et al Zeezoogdieren in de Eems: studie naar de effecten van bouwactiviteiten van GSP, RWE en NUON in de Eemshaven in 2010 Den Burg: IMARES; 2011.

[73] Kirkwood RJ, Aarts GM, Brasseur SMJM. Seal monitoring and evaluation for the Luchterduinen offshore wind farm: 2 T-construction—2014 report. Den Helder: IMARES; 2015.

[74] Brasseur SMJM, Kirkwood RJ. Seal monitoring and evaluation for the Gemini offshore windpark: T-construction—2015 report. Den Burg: IMARES; 2016. Report No.: C043/16.

[75] Tougaard J, Tougaard S, Jensen RC, Jensen T, Teilmann J, Adelung D, et al Harbour seals on Horns Reef before, during and after construction of Horns Rev Wind Farm.; 2006.

[76] Waterbolk HT. Oude bewoning in het Waddengebied In: Abrahamse WJ J., van Leeuwen-Seelt N., editor. Waddenzee. Harlingen Amsterdam: Landelijke Vereniging tot Behoud van de Waddenzee Natuurmonumenten; 1976 p. 211–21.

[77] Hart tP. Zeehondenjacht in Nederland 1591–1962 (*Seal hunting in the Netherlands*, *1591–1962*) Amsterdam: Free University Amsterdam; 2007.

[78] Bemmel ACVv. Planning a census of the harbour seal (*Phoca vitulina L*.) on the coasts of the Netherlands. Beaufortia 1956;5(54):121–32.

[79] Hoffmeyer H. Seehunde und Seehundsjagd an der deutschen Nordseeküste. Zeitschrift für Jagdwissenschaft. 1962;8(1):1–13.

[80] Härkönen T, Harding KC. Spatial structure of harbour seal populations and the implications thereof. Canadian Journal of Zoology-Revue Canadienne De Zoologie. 2001;79(12):2115–27.

[81] Dietz R, Teilmann J, Andersen SM, Rigét F, Olsen MT. Movements and site fidelity of harbour seals (*Phoca vitulina*) in Kattegat, Denmark, with implications for the epidemiology of the phocine distemper virus. ICES Journal of Marine Science. 2012;70(1):186–95.

[82] Womble JN, Gende SM. Post-breeding season migrations of a top predator, the harbor seal (*Phoca vitulina richardii*), from a marine protected area in Alaska. PLoS ONE. 2013;8(2):e55386 doi: 10.1371/journal.pone.0055386 23457468 10.1371/journal.pone.0055386 PMC3573017

[83] Sharples RJ, Moss SE, Patterson TA, Hammond PS. Spatial variation in foraging behaviour of a marine top predator (*Phoca vitulina*) determined by a large-scale satellite tagging program. PLoS one. 2012;7(5):e37216 doi: 10.1371/journal.pone.0037216 22629370 10.1371/journal.pone.0037216 PMC3357409

[84] Contasti AL, Van Beest FM, Vander Wal E, McLoughlin PD. Identifying hidden sinks in growing populations from individual fates and movements: The feral horses of Sable Island. The Journal of Wildlife Management. 2013;77(8):1545–52.

[85] Gundersen G, Johannesen E, Andreassen HP, Ims RA. Source–sink dynamics: how sinks affect demography of sources. Ecology Letters. 2001;4(1):14–21.

