# Supplementary material for: Echoes from the past: Regional variations in recovery within a harbour seal population
Source: PLoS One. 2018 Jan 3;13(1):e0189674. doi: 10.1371/journal.pone.0189674 (PMC5751996; doi:10.1371/journal.pone.0189674)
Supplement: S1 Table — M = moult, P = pupping season. (DOCX) [file pone.0189674.s001.docx]

**S1 Table**. Overview of number of surveys per year. M = moult, P = pupping season

|  | **The Netherlands** | | | **Lower Saxony** | | | **Schleswig-Holstein** | | | **Denmark** | | |
| --- | --- | --- | --- | --- | --- | --- | --- | --- | --- | --- | --- | --- |
|  | **M** | **P** | **Total** | **M** | **P** | **Total** | **M** | **P** | **Total** | **M** | **P** | **Total** |
| **1974** | 2 | 2 | 4 | 1* | 1* |  | 1* | 1* |  | 1* | 1* |  |
| **1975** | 2 | 7 | 9 | 1* | 1* |  | 1* | 1* |  | 1* | 1* |  |
| **1976** | 3 | 6 | 9 | 1* | 1* |  | 1* | 1* |  | 1* | 1* |  |
| **1977** | 2 | 6 | 8 | 1* | 1* |  | 1* | 1* |  | 1* | 1* |  |
| **1978** | 3 | 5 | 8 | 1* | 1* |  | 1* | 1* |  | 1* | 1* |  |
| **1979** | 2 | 4 | 6 | 1* | 1* |  | 1* | 1* |  | 1* | 1* |  |
| **1980** | 2 | 5 | 7 | 1* | 1* |  | 1* | 1* |  | 1* | 1* |  |
| **1981** | 2 | 5 | 7 | 1* | 1* |  | 1* | 1* |  | 1* | 1* |  |
| **1982** | 1 | 6 | 7 | 1* | 1* |  | 1* | 1* |  | 1* | 1* |  |
| **1983** | 1 | 5 | 6 | 1* | 1* |  | 1* | 1* |  | 1* | 1* |  |
| **1984** | 1 | 5 | 6 | 1* | 1* |  | 1* | 1* |  | 1* | 1* |  |
| **1985** | 2 | 4 | 6 | 1* | 1* |  | 1* | 1* |  | 1* | 1* |  |
| **1986** | 2 | 5 | 7 | 1* | 1* |  | 1* | 1* |  | 1* | 1* |  |
| **1987** | 2 | 6 | 8 | 1* | 1* |  | 1* | 1* |  | 1* | 1* |  |
| **1989** | 3 | 6 | 9 | 3 | 7 | 10 | 3 | 6 | 9 | 1 | 2 | 3 |
| **1990** | 3 | 6 | 9 | 1 | 5 | 6 | 2 | 5 | 7 | 2 | 5 | 7 |
| **1991** | 2 | 6 | 8 | 2 | 8 | 10 | 3 | 5 | 8 | 1 | 7 | 8 |
| **1992** | 2 | 7 | 9 | 2 | 8 | 10 | 2 | 5 | 7 | 2 | 6 | 8 |
| **1993** | 2 | 7 | 9 | 2 | 7 | 9 | 1 | 5 | 6 | 3 | 5 | 8 |
| **1994** | 2 | 6 | 8 | 1 | 6 | 7 | 1 | 7 | 8 | 1 | 4 | 5 |
| **1995** | 2 | 4 | 6 | 1 | 5 | 6 | 2 | 4 | 6 | 1 | 2 | 3 |
| **1996** | 1 | 3 | 4 |  | 5 | 5 | 2 | 2 | 4 | 2 | 1 | 3 |
| **1997** | 2 | 5 | 7 | 2 | 6 | 8 | 2 | 4 | 6 | 2 | 2 | 4 |
| **1998** | 2 | 3 | 5 | 2 | 3 | 5 | 1 | 2 | 3 | 1 | 2 | 3 |
| **1999** | 2 | 3 | 5 | 2 | 3 | 5 | 1 | 3 | 4 | 3 | 1 | 4 |
| **2000** | 2 | 4 | 6 | 2 | 5 | 7 | 2 | 3 | 5 | 2 | 3 | 5 |
| **2001** | 1 | 3 | 4 | 2 | 3 | 5 | 2 | 3 | 5 | 2 | 2 | 4 |
| **2003** | 2 | 3 | 5 | 2 | 3 | 5 | 2 | 3 | 5 | 2 | 2 | 4 |
| **2004** | 1 | 3 | 4 | 2 | 3 | 5 | 2 | 2 | 4 | 2 | 2 | 4 |
| **2005** | 2 | 2 | 4 | 2 | 3 | 5 | 2 | 3 | 5 | 1 | 3 | 4 |
| **2006** | 2 | 3 | 5 | 2 | 3 | 5 | 2 | 2 | 4 | 1 | 3 | 4 |
| **2007** | 2 | 3 | 5 | 2 | 2 | 4 | 2 | 3 | 5 | 2 | 2 | 4 |
| **2008** | 2 | 3 | 5 |  | 2 | 2 | 2 | 3 | 5 | 2 | 3 | 5 |
| **2009** | 2 | 3 | 5 | 1 | 3 | 4 | 2 | 3 | 5 | 2 | 3 | 5 |
| **2010** | 2 | 2 | 4 | 2 | 2 | 4 | 2 | 3 | 5 | 2 | 3 | 5 |
| **2011** | 2 | 2 | 4 | 1 | 3 | 4 | 1 | 3 | 4 | 2 | 3 | 5 |
| **2012** | 2 | 3 | 5 | 2 | 3 | 5 | 2 | 2 | 4 | 2 | 3 | 5 |
| **2013** | 1 | 3 | 4 | 2 | 2 | 4 | 2 | 1 | 3 | 2 | 3 | 5 |
| **2014** | 3 | 4 | 7 | 2 | 3 | 5 | 2 | 3 | 5 | 2 | 3 | 5 |

*Only data for maximum counts of that year were available
