# Supplementary material for: Echoes from the past: Regional variations in recovery within a harbour seal population
Source: PLoS One. 2018 Jan 3;13(1):e0189674. doi: 10.1371/journal.pone.0189674 (PMC5751996; doi:10.1371/journal.pone.0189674)
Supplement: S2 Table — Left column indicates the periods. (DOCX) [file pone.0189674.s002.docx]

**S2 Table.** Annual maximum counts for total numbers (during moult) and pups. Left column indicates the periods.

|  |  | **Denmark** | | **Schleswig-Holstein** | | **Lower Saxony** | | **The Netherlands** | |
| --- | --- | --- | --- | --- | --- | --- | --- | --- | --- |
| **period** | **year** | **TOTAL** | **PUPS** | **TOTAL** | **PUPS** | **TOTAL** | **PUPS** | **TOTAL** | **PUPS** |
| **I** | **1974** | 350 | 41 | 1544 | 377 | 1127 | 215 | 569 | 54 |
| **I** | **1975** | 360 | 59 | 1749 | 403 | 1019 | 200 | 509 | 82 |
| **I** | **1976** | 389 | 64 | 1653 | 404 | 1165 | 253 | 481 | 71 |
| **I** | **1977** | 410 | 67 | 1806 | 488 | 1131 | 198 | 463 | 54 |
| **I** | **1978** | 332 | 53 | 1795 | 469 | 1199 | 232 | 458 | 78 |
| **I** | **1979** | 421 | 110 | 1919 | 417 | 1109 | 168 | 543 | 54 |
| **I** | **1980** | 671 | 140 | 2202 | 481 | 1310 | 230 | 514 | 61 |
| **I** | **1981** | 656 | 150 | 2200 | 461 | 1458 | 246 | 578 | 69 |
| **I** | **1982** | 789 | 130 | 2300 | 504 | 1569 | 254 | 654 | 88 |
| **I** | **1983** | 924 | 152 | 2500 | 547 | 1789 | 337 | 712 | 87 |
| **I** | **1984** | 853 | 141 | 2700 | 589 | 1630 | 270 | 738 | 83 |
| **I** | **1985** | 958 | 158 | 3300 | 750 | 2062 | 324 | 775 | 101 |
| **I** | **1986** | 1261 | 208 | 3195 | 641 | 2272 | 389 | 798 | 101 |
| **I** | **1987** | 1477 | 243 | 3793 | 882 | 2400 | 427 | 1051 | 154 |
| **II** | **1989** | 869 | 94 | 1558 | 191 | 1401 | 229 | 533 | 108 |
| **II** | **1990** | 1048 | 172 | 1786 | 391 | 1458 | 344 | 559 | 122 |
| **II** | **1991** | 1097 | 193 | 2132 | 411 | 1977 | 481 | 750 | 138 |
| **II** | **1992** | 1168 | 214 | 2608 | 541 | 2246 | 482 | 957 | 178 |
| **II** | **1993** | 1433 | 222 | 3118 | 540 | 2457 | 555 | 1074 | 198 |
| **II** | **1994** | 1507 | 259 | 3086 | 580 | 3078 | 647 | 1230 | 227 |
| **II** | **1995** | 1610 | 261 | 3527 | 707 | 3184 | 583 | 1410 | 261 |
| **II** | **1996** | 1634 | 353 | 4260 | 830 | 3489 | 713 | 1690 | 290 |
| **II** | **1997** | 1924 | 380 | 4664 | 1084 | 4272 | 909 | 2020 | 405 |
| **II** | **1998** | 2300 | 359 | 5278 | 1071 | 4529 | 795 | 2280 | 477 |
| **II** | **1999** | 2183 | 313 | 5853 | 1358 | 4725 | 920 | 2399 | 511 |
| **II** | **2000** | 2145 | 389 | 6300 | 1540 | 5167 | 1067 | 3330 | 594 |
| **II** | **2001** | 2380 | 396 | 7190 | 1727 | 6092 | 1060 | 3594 | 765 |
| **III** | **2003** | 1256 | 270 | 5038 | 1407 | 3393 | 799 | 2366 | 480 |
| **III** | **2004** | 1479 | 283 | 6044 | 1781 | 3968 | 933 | 3194 | 694 |
| **III** | **2005** | 1899 | 388 | 6762 | 2046 | 4766 | 1166 | 3531 | 897 |
| **III** | **2006** | 2216 | 411 | 7160 | 2085 | 4574 | 1157 | 4065 | 850 |
| **III** | **2007** | 2499 | 341 | 7416 | 2095 | 4550 | 594 | 4572 | 978 |
| **III** | **2008** | 2656 | 484 | 8352 | 2096 | 6030 | 1035 | 5972 | 976 |
| **III** | **2009** | 3063 | 490 | 8415 | 2263 | 6226 | 1421 | 6399 | 1313 |
| **III** | **2010** | 2909 | 564 | 9720 | 2873 | 6395 | 1605 | 6181 | 1451 |
| **III** | **2011** | 3386 | 699 | 10941 | 3294 | 7163 | 1517 | 7378 | 1445 |
| **III** | **2012** | 3966 | 570 | 11262 | 3247 | 8029 | 1876 | 7328 | 1473 |
| **III** | **2013** | 3133 | 613 | 11892 | 3682 | 8082 | 1373 | 7605 | 1403 |
| **III** | **2014** | 3368 | 654 | 13420 | 3853 | 9343 | 2067 | 7356 | 1942 |
